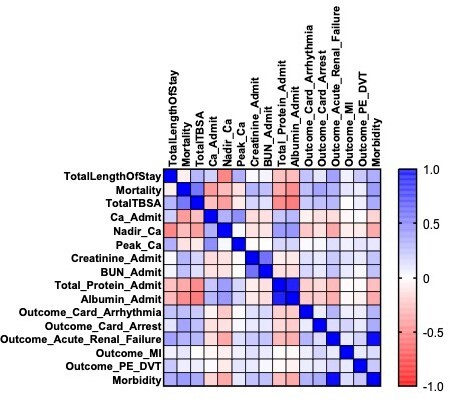# 597 Hypercalcemia in Burn Intensive Care: A Retrospective Analysis with Implications for Patient Outcome Prediction

**DOI:** 10.1093/jbcr/irae036.231

**Published:** 2024-04-17

**Authors:** Vishal Bandaru, Tristin Chaudhury, Hannah Chaudhury, Vivie Tran, Shakira Meltan, Caezaan Keshvani, Alan Pang

**Affiliations:** Texas Tech University Health Sciences Center, Austin, TX; Texas Tech Health Sciences Center, Lubbock, TX; Texas Tech University Health Sciences Center School of Medicine, Lubbock, TX; TTUHSC, Lubbock, TX; Texas Tech University Health Sciences Center, Lubbock, TX; Texas Tech University Health Sciences Center, Austin, TX; Texas Tech Health Sciences Center, Lubbock, TX; Texas Tech University Health Sciences Center School of Medicine, Lubbock, TX; TTUHSC, Lubbock, TX; Texas Tech University Health Sciences Center, Lubbock, TX; Texas Tech University Health Sciences Center, Austin, TX; Texas Tech Health Sciences Center, Lubbock, TX; Texas Tech University Health Sciences Center School of Medicine, Lubbock, TX; TTUHSC, Lubbock, TX; Texas Tech University Health Sciences Center, Lubbock, TX; Texas Tech University Health Sciences Center, Austin, TX; Texas Tech Health Sciences Center, Lubbock, TX; Texas Tech University Health Sciences Center School of Medicine, Lubbock, TX; TTUHSC, Lubbock, TX; Texas Tech University Health Sciences Center, Lubbock, TX; Texas Tech University Health Sciences Center, Austin, TX; Texas Tech Health Sciences Center, Lubbock, TX; Texas Tech University Health Sciences Center School of Medicine, Lubbock, TX; TTUHSC, Lubbock, TX; Texas Tech University Health Sciences Center, Lubbock, TX; Texas Tech University Health Sciences Center, Austin, TX; Texas Tech Health Sciences Center, Lubbock, TX; Texas Tech University Health Sciences Center School of Medicine, Lubbock, TX; TTUHSC, Lubbock, TX; Texas Tech University Health Sciences Center, Lubbock, TX; Texas Tech University Health Sciences Center, Austin, TX; Texas Tech Health Sciences Center, Lubbock, TX; Texas Tech University Health Sciences Center School of Medicine, Lubbock, TX; TTUHSC, Lubbock, TX; Texas Tech University Health Sciences Center, Lubbock, TX

## Abstract

**Introduction:**

Burn injuries account for significant acute hospitalizations in the US and are a global concern with approximately 180,000 deaths annually. These injuries elicit drastic changes in serum calcium levels due to physiological systems and factors such as imbalances in bone formation/resorption, Acute Renal Failure (ARF), burn severity (BSA) and patient immobilization. This study aims to explore the multifaceted causes of hypercalcemia in burn patients at our university hospital. We conducted a preliminary study of n= 200 burn patients and noted a strong positive correlation between peak calcium with acute renal failure, cardiac arrest, and TBSA (total body surface area).

We hypothesize that patients with higher burn severity, longer hospitalization, and renal complications will experience a more significant increase in serum calcium. Additionally, patients with hypercalcemia are expected to have higher morbidity and mortality rates than those with normal calcium levels.

**Methods:**

This was a retrospective hypothesis-driven study of n= 832 burn patients that aims to analyze calcium levels by utilizing our hospital burn registry and Electronic Medical Record to gather pertinent data.

**Results:**

After adjusting for age, comorbidities, and TBSA percentage, there was a strong negative correlation between nadir calcium and arrhythmia (r = -0.21), renal failure (r = -0.34), and morbidity (r = -0.34). There was no correlation between peak calcium and these three categories mentioned. Nadir calcium was negatively correlated with total length of stay (r = -0.47), mortality (r = -0.26), and total TBSA (r = -0.38). In addition, admission calcium levels had a strong positive correlation with nadir (r= 0.31) and peak calcium (r= 0.44) levels.

**Conclusions:**

Compared to the preliminary study, a positive correlation was not observed between peak calcium and arrythmia, renal failure, and total TBSA. This data gathered emphasized nadir calcium as a potential marker for patient outcomes for admitted burn-related injuries. In addition, nadir calcium’s strong correlation with admission calcium suggests large fluctuations between these variables could help assess patient outcomes.

**Applicability of Research to Practice:**

This study suggests potential connections between burn-related injuries and nadir calcium levels, underscoring the necessity for further research and rigorous statistical analysis. These findings could potentially prompt the adoption of nadir calcium as an admission marker for detecting potential adverse effects in clinical practice.